# Orchestration of Photosynthesis-Associated Gene Expression and Galactolipid Biosynthesis during Chloroplast Differentiation in Plants

**DOI:** 10.1093/pcp/pcae049

**Published:** 2024-04-26

**Authors:** Sho Fujii, Hajime Wada, Koichi Kobayashi

**Affiliations:** Department of Biology, Faculty of Agriculture and Life Science, Hirosaki University, 3 Bunkyo-cho, Hirosaki, Aomori, 036-8561 Japan; Department of Life Sciences, Graduate School of Arts and Sciences, The University of Tokyo, 3-8-1 Komaba, Meguro-ku, Tokyo, 153-8902 Japan; Department of Biology, Graduate School of Science, Osaka Metropolitan University, 1-1 Gakuen-cho, Naka-ku, Sakai, Osaka, 599-8531 Japan; Faculty of Liberal Arts, Science and Global Education, Osaka Metropolitan University, 1-1 Gakuen-cho, Naka-ku, Sakai, Osaka, 599-8531 Japan

**Keywords:** *Arabidopsis thaliana*, Digalactosyldiacylglycerol, Monogalactosyldiacylglycerol, Plastid, Retrograde signaling, Thylakoid membrane

## Abstract

The chloroplast thylakoid membrane is composed of membrane lipids and photosynthetic protein complexes, and the orchestration of thylakoid lipid biosynthesis and photosynthesis-associated protein accumulation is considered important for thylakoid development. Galactolipids consist of ∼80% of the thylakoid lipids, and their biosynthesis is fundamental for chloroplast development. We previously reported that the suppression of galactolipid biosynthesis decreased the expression of photosynthesis-associated nuclear-encoded genes (**PhAPGs**) and photosynthesis-associated plastid-encoded genes (**PhAPGs**). However, the mechanism for coordinative regulation between galactolipid biosynthesis in plastids and the expression of PhANGs and PhAPGs remains largely unknown. To elucidate this mechanism, we investigated the gene expression patterns in galactolipid-deficient ***Arabidopsis*** seedlings during the de-etiolation process. We found that galactolipids are crucial for inducing both the transcript accumulation of PhANGs and PhAPGs and the accumulation of plastid-encoded photosynthesis-associated proteins in developing chloroplasts. Genetic analysis indicates the contribution of the GENOMES UNCOUPLED1 (GUN1)–mediated plastid-to-nucleus signaling pathway to PhANG regulation in response to galactolipid levels. Previous studies suggested that the accumulation of GUN1 reflects the state of protein homeostasis in plastids and alters the PhANG expression level. Thus, we propose a model that galactolipid biosynthesis determines the protein homeostasis in plastids in the initial phase of de-etiolation and optimizes GUN1-dependent signaling to regulate the PhANG expression. This mechanism might contribute to orchestrating the biosynthesis of lipids and proteins for the biogenesis of functional chloroplasts in plants.

## Introduction

Chloroplast biogenesis requires gene expression in both nuclei and plastids, as chloroplast protein complexes such as photosystem I (PSI), photosystem II (PSII), ATP synthase and ribosomes are composed of subunits encoded in nuclear and plastid genomes. Bidirectional communication between nuclei and plastids is considered important for coordinative regulation of gene expression in these two organelles. Many photosynthesis-associated plastid-encoded genes (PhAPGs) are transcribed by the plastid-encoded RNA polymerase (PEP), which comprises α-, β-, β'- and β”-subunits encoded by *rpoA, rpoB, rpoC1* and *rpoC2* genes, respectively (Pfannschmidt et al. 2015, Ortelt and Link 2021). Transcription of the *rpo* genes depends on the nuclear-encoded RNA polymerase (NEP) targeted to plastids (Pfannschmidt et al. 2015, Ortelt and Link 2021). In addition to the PEP core subunits, sigma factors (SIGs) encoded in the nuclear genome are required for PEP activity, and thus plastid gene expression is regulated by the nucleus (Shiina et al. 2005, Lysenko 2007). On the other hand, the condition of plastids largely influences nuclear gene expression. Inhibitors and mutations that disrupt chloroplast functionality strongly downregulate photosynthesis-associated nuclear-encoded genes (PhANGs) (Oelmüller et al. 1986, Oelmüller and Mohr 1986, Susek et al. 1993, Sullivan and Gray 1999, Moulin et al. 2008, Ruckle et al. 2012). Such a mechanism is called plastid-to-nucleus retrograde signaling or plastid signaling. Metabolism of tetrapyrrole and isoprenoids, plastid gene expression, redox state and the accumulation of reactive oxygen species are known as sources of plastid signaling (Nott et al. 2006, Chan et al. 2016, Wu and Bock 2021).

*GENOMES UNCOUPLED1* (*GUN1*) was isolated as one of the genes involved in plastid signaling (Susek et al. 1993). The loss of GUN1 protein attenuates the downregulation of PhANG expression in response to the inhibition of chloroplast gene expression by rifampicin and lincomycin (Susek et al. 1993). Further analyses revealed involvements of GUN1 in plastid signaling triggered by mutations of genes for transcription (Woodson et al. 2013), translation (Tadini et al. 2016, Marino et al. 2019), protein homeostasis (Kakizaki et al. 2009, Wu et al. 2018), sugar metabolism (Maruta et al. 2015) and other several processes in plastids (Wu and Bock 2021). *GUN1* encodes a pentatricopeptide repeat protein targeted to plastids (Koussevitzky et al. 2007) and interacts with proteins involved in tetrapyrrole metabolism, transcription and RNA editing, in addition to ribosomal proteins and plastid chaperons (Tadini et al. 2016, Zhao et al. 2019), suggesting that GUN1 monitors the condition of tetrapyrrole metabolism, gene expression and protein homeostasis in plastids (Pesaresi and Kim 2019, Shimizu and Masuda 2021). GUN1 proteins are subjected to rapid turnover by the Clp proteases in functional chloroplasts (Wu et al. 2018), leading to low accumulation levels of GUN1. Disturbance of chloroplast functionality slows down the degradation of GUN1 and consequently activates repressive plastid signaling (Wu et al. 2018). A recent report demonstrated that GUN1 is a heme-binding protein involved in the regulation of tetrapyrrole metabolism and the accumulation of GUN1 proteins decreased the abundance of heme (Shimizu et al. 2019). As heme is known to accelerate PhANG expression even under stressful conditions for chloroplasts (Woodson et al. 2011), GUN1 accumulated in response to perturbation of chloroplast functions may downregulate the PhANG expression by regulating heme homeostasis. GOLDEN2-LIKE (GLK) transcription factors are known to induce the expression of PhANGs and thereby chloroplast biogenesis (Fitter et al. 2002, Waters et al. 2009). A previous study showed that GUN1 negatively regulates the *GLK* gene expression upon chloroplast dysfunction (Tokumaru et al. 2017), suggesting an involvement of GLKs in PhANG regulation in response to plastid signaling.

Synthesis of thylakoid membrane lipids is critical for chloroplast development. Monogalactosyldiacylglycerol (MGDG) and digalactosyldiacylglycerol (DGDG) constitute ∼50 and ∼30% of the thylakoid lipids, respectively (Dorne et al. 1990). In *Arabidopsis* chloroplasts, the bulk of MGDG is synthesized by inner envelope-targeted MGDG synthase (MGD) 1, which transfers galactose from UDP-galactose to diacylglycerol (Awai et al. 2001, Kobayashi et al. 2007, 2009). DGDG synthase (DGD) 1, which adds another galactose moiety to MGDG in the plastid outer envelope, contributes to DGDG biosynthesis predominantly in photosynthetic tissues (Dörmann et al. 1995, Kelly et al. 2003). Abolished accumulation of MGDG and DGDG by knock-out mutation of *MGD1* (*mgd1-2*) or inducible suppression of *MGD1* mediated by dexamethasone (DEX)-inducible microRNA (*amiR-MGD1*) severely impaired the thylakoid formation, chlorophyll accumulation and expression of both PhANGs and PhAPGs in *Arabidopsis* (Kobayashi et al. 2007, 2013, Fujii et al. 2014). Decreased accumulation of MGDG by knock-down mutation of *MGD1* also impaired chloroplast development and chlorophyll accumulation in *Arabidopsis* and tobacco (Jarvis et al. 2000, Wu et al. 2013). A knock-out mutation of *DGD1* (*dgd1-1*) substantially decreased the content of DGDG but not MGDG and resulted in attenuated thylakoid formation and chlorophyll accumulation particularly in the de-etiolation process (Dörmann et al. 1995, Fujii et al. 2019). These findings suggest that both MGDG and DGDG are crucial for chloroplast biogenesis. Recently, we reported that these galactolipids are involved in the chlorophyll biosynthesis pathway and the light-induced PhANG expression (Fujii et al. 2017, 2018, 2019).

Phosphatidylglycerol (PG) accounts for ∼10% of the thylakoid lipids and is also essential for chloroplast development (Hagio et al. 2002). Loss of plastidic PG biosynthesis led to a strong impairment of chlorophyll accumulation and expression of PhANGs and PhAPGs (Kobayashi et al. 2015). The majority of PhANGs regulated by GLKs were downregulated in the PG-deficient mutant, and overexpression of *GLK1* increased the PhANG expression in PG-deficient seedlings (Fujii et al. 2022). These results suggest the contribution of GLK-mediated plastid signaling to balancing PG biosynthesis in plastids and PhANG expression. Moreover, increased expression of *GLK1* did not recover the PhAPG expression in PG-less plants, indicating the tight relationship between PG biosynthesis and/or subsequent thylakoid formation and PhAPG expression (Fujii et al. 2022). By contrast, it remains elusive how galactolipid biosynthesis and photosynthesis-associated gene expression are coordinatively regulated.

To address the mechanism for the orchestration of galactolipid biosynthesis and photosynthesis-associated gene expression, we examined the transcriptional profiles and protein accumulation patterns of *MGD1*-suppressed seedlings in the de-etiolation process. By introducing the *gun1* mutation in *amiR-MGD1* transgenic lines, we demonstrated that GUN1-mediated plastid signaling contributes to the coordination of galactolipid biosynthesis and PhANG expression. Comparison between the *amiR-MGD1* lines, *dgd1-1* mutant and chlorophyll-deficient mutants revealed the particular importance of galactolipid biosynthesis in PhAPG expression.

## Results

### Expression of PhAPGs was repressed in galactolipid-deficient seedlings

**Fig. 1:**
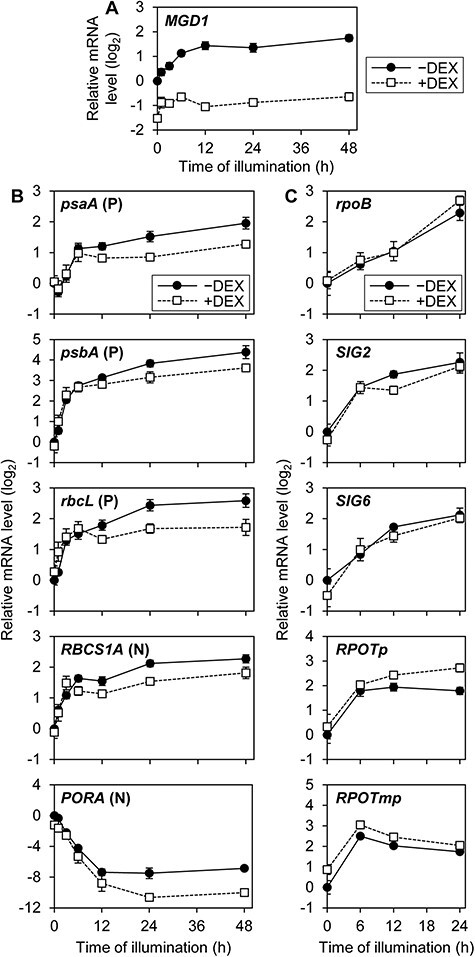
mRNA accumulation during de-etiolation in *amiR-MGD1* seedlings. (A) RT-qPCR analysis of *MGD1*. Data were adapted from a previous article (Fujii et al. 2019) as a reference. (B) RT-qPCR analyses of PhAPGs (P) and PhANGs (N). (C) RT-qPCR analyses of genes involved in plastid gene expression. In (A–C), seedlings were illuminated under continuous light for the indicated time length after 4-day growth in the dark (0 h). +DEX indicates seedlings supplemented with DEX from germination until being harvested, whereas −DEX indicates non-treated controls. Transcript levels were normalized to *ACTIN8* and presented as the difference from the control before illumination. Data are means ± standard errors (SE) from 13 (*MGD1* and *PORA*, 0 h) or three (others) biological replicates.

We previously reported that DEX treatment on *amiR-MGD1* seedlings suppressed *MGD1* expression throughout the de-etiolation process (**Fig. 1A**) and diminished MGDG accumulation (Fujii et al. 2019). By the *MGD1* suppression in this transgenic line, transcript accumulation of PhANGs, namely, *HEMA1* encoding the major isoform of glutamyl-tRNA^Glu^ reductase, *CHLH* encoding the H subunit of Mg-chelatase and *LHCB1* and *LHCB6* encoding LHCII subunits, was decreased after illumination of etiolated seedlings (Fujii et al. 2019). To elucidate the effect of MGDG deficiency in PhAPGs, we measured the mRNA level of *psaA, psbA* and *rbcL*, encoding the PsaA subunit of PSI, the D1 subunit of PSII and the large subunit of Rubisco, respectively, during the process of de-etiolation in *amiR-MGD1* (**Fig. 1B**). We also analyzed the mRNA profile of *RBCS1A*, which is the nuclear gene encoding a major isoform of Rubisco small subunits. In control seedlings (−DEX), transcripts of PhAPGs accumulated rapidly during the first 6 h of illumination and gradually increased afterward. *MGD1* suppression by DEX treatment (+DEX) did not affect the rapid induction within 6 h but repressed the later gradual increases. *RBCS1A* represented a similar but milder decrease compared to other PhANGs (Fujii et al. 2019). The transcript level of *PORA*, which encodes an isoform of light-dependent NADPH:protochlorophyllide oxidoreductase, decreased after light illumination in DEX-untreated seedlings, as described previously in wild-type plants (Armstrong et al. 1995). The mRNA abundance of this gene was smaller in *MGD1*-suppressed seedlings than in the control throughout the de-etiolation process. Combined with our previous data (Fujii et al. 2019), these results indicate that the expression of both PhANGs and PhAPGs requires MGDG biosynthesis during chloroplast development.

To investigate the mechanism underlying the repression of PhAPG expression in *MGD1*-suppressed seedlings, we tested the mRNA levels of genes involved in PhAPG expression (**Fig. 1C**). *SIG2* and *SIG6* encode plastid SIG isoforms, which are important for PEP activity during chloroplast biogenesis (Woodson et al. 2013). *MGD1* suppression did not affect the transcript accumulation of *SIG2* and *SIG6* as well as *rpoB*. By contrast, mRNA levels of *RPOTp* and *RPOTmp*, which encode NEP isoforms targeted to plastids specifically and to both mitochondria and plastids, respectively (Liere et al. 2011), were slightly increased by the suppression of *MGD1*, probably due to the compensatory effects of downregulated PhAPG expression. These data imply that the decreased mRNA accumulation of PhAPGs in MGDG-deficient seedlings was not caused by the downregulation of genes involved in plastid gene expression.

### Suppression of chlorophyll biosynthesis had a smaller impact on photosynthesis-associated gene expression than galactolipid deficiency

**Fig. 2:**
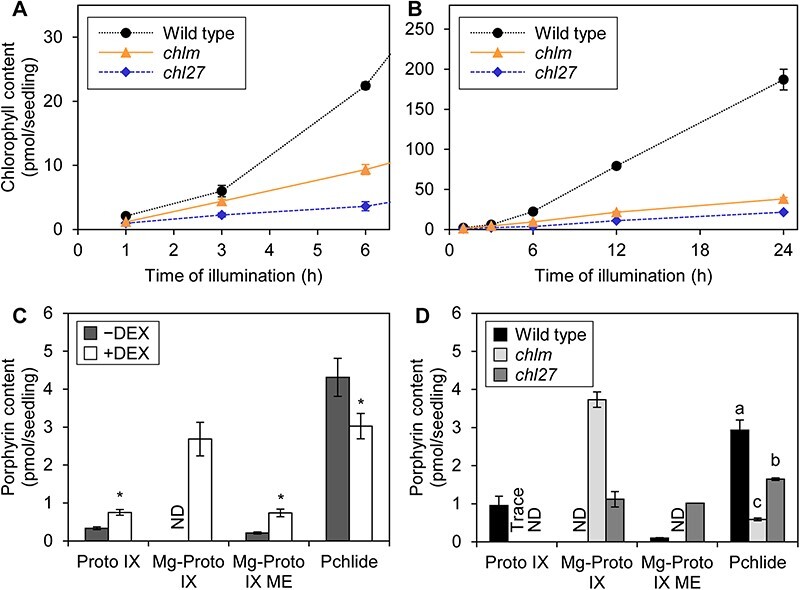
Chlorophyll biosynthesis in *amiR-MGD1, chlm* and *chl27* seedlings. (A and B) Chlorophyll accumulation during de-etiolation. (A) The initial phase of de-etiolation. Seedlings were illuminated under continuous light for the indicated time length after 4-day growth in the dark. Data are means ± SE from three to six biological replicates. (C and D) Accumulation of chlorophyll intermediates in seedlings illuminated for 3 h after 4-day growth in the dark. After 3 h of illumination, seedlings were supplemented with ALA for 1 h in the dark. Data are means ± SE from three biological replicates. In (C), +DEX indicates *amiR-MGD1* seedlings supplemented with DEX from germination until being harvested, whereas −DEX indicates non-treated controls. Asterisks indicate statistical significance (*P* < 0.05, Student’s *t*-test). In (D), different letters indicate statistical significance (*P* < 0.05, Tukey–Kramer’s multiple comparison test). ND, not detected; Trace, trace amount.

To examine if the downregulation of PhANGs and PhAPGs observed in *amiR-MGD1* is specific to MGDG deficiency, we compared this transgenic line with mutants deficient in chlorophyll biosynthesis. *chlm* and *chl27* are knock-down mutants with T-DNA insertion in the 5ʹ-UTR of genes encoding Mg-protoporphyrin (Mg-Proto) IX methyltransferase and Mg-Proto IX monomethylester (ME) cyclase, respectively (Bang et al. 2008, Mochizuki et al. 2008). Chlorophyll accumulation in *chlm* and *chl27* was substantially repressed during de-etiolation, which resulted in 88 and 80% lower chlorophyll content in *chlm* and *chl27*, respectively, than in the wild type at 24 h of illumination (**Fig. 2A, B**). The decreases in chlorophyll content in these mutants were comparable to those in DEX-treated *amiR-MGD1* (87% lower than the DEX-untreated control at 24 h of illumination) (Fujii et al. 2019). To assess the state of the chlorophyll biosynthesis pathway in illuminated seedlings, etiolated seedlings were exposed to light for 3 h and then supplemented with the tetrapyrrole precursor 5-aminolevulinic acid (ALA) for 1 h in darkness. DEX-treated *amiR-MGD1* accumulated larger amounts of chlorophyll intermediates, namely, Proto IX, Mg-Proto IX and Mg-Proto IX ME compared to the control seedlings, whereas the level of protochlorophyllide (Pchlide) was slightly decreased (**Fig. 2C**). The data are similar to those observed in etiolated seedlings of DEX-treated *amiR-MGD1* (Fujii et al. 2017), indicating that the chlorophyll biosynthesis pathway is continuously impaired by loss of MGDG biosynthesis throughout etiolated and de-etiolated growth. Similarly, *chlm* and *chl27* mutants illuminated for 3 h showed lower Pchlide accumulation with 1 h ALA treatment in the dark than the wild-type control (**Fig. 2D**). In addition, *chlm* showed excess levels of Mg-Proto IX, whereas *chl27* showed high accumulation of both Mg-Proto IX and Mg-Proto IX ME. Thus, in both DEX-treated *amiR-MGD1* and the chlorophyll biosynthesis mutants, the impaired metabolism of chlorophyll intermediates would be the main cause of attenuated chlorophyll accumulation during the initial phase of de-etiolation.

**Fig. 3:**
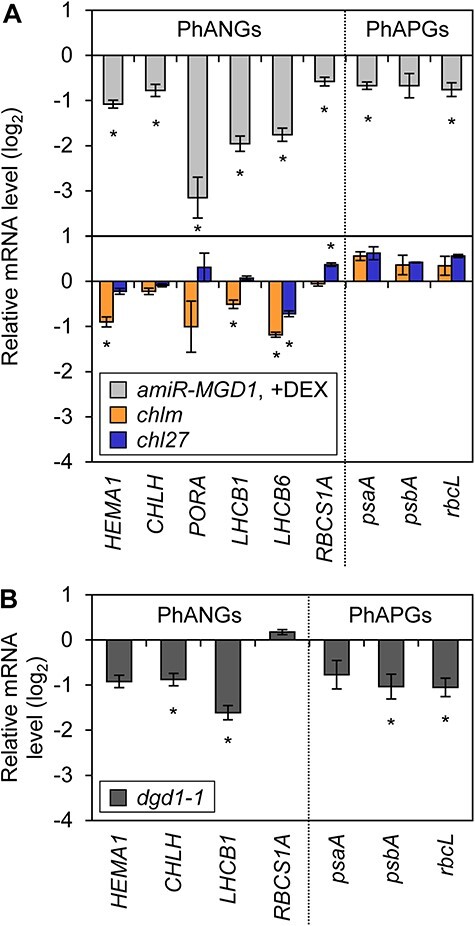
Transcript accumulation of PhANGs and PhAPGs in *amiR-MGD1, chlm, chl27* and *dgd1-1* seedlings. (A) *amiR-MGD1* and chlorophyll biosynthesis mutants *chlm* and *chl27*. (B) DGDG-deficient *dgd1-1* mutants. In (A) and (B), seedlings were illuminated under continuous light for 24 h after 4-day growth in the dark. Transcript levels were normalized to *ACTIN8* and presented as the difference from each control (wild type or −DEX control). Data of *amiR-MGD1* were adapted from a previous article (Fujii et al. 2019) and **Fig. 1** for comparison. Asterisks indicate statistically significant differences from each control (*P* < 0.05, Student’s *t*-test for *amiR-MGD1* and *dgd1-1* and Tukey–Kramer’s multiple comparison test for *chlm* and *chl27*).

Because the *chlm* and *chl27* mutants showed impaired chlorophyll synthesis similar to DEX-treated *amiR-MGD1* during de-etiolation, we tested whether they exhibit impairments in the expression of PhANGs and PhAPGs as observed in *MGD1*-suppressed seedlings. In *chlm*, mRNA accumulation of three of the six PhANGs tested, namely *HEMA1, LHCB1* and *LHCB6*, was slightly attenuated after light illumination of etiolated seedlings, but the profiles of other genes (*CHLH, PORA* and *RBCS1A*) were similar to those in the wild type (**Supplementary Fig. S1**). The knock-down mutation of *CHL27* did not downregulate PhAPGs except *LHCB6*. Neither *chlm* nor *chl27* showed decreased mRNA accumulation of the three PhAPGs (*psaA, psbA* and *rbcL*) tested. After 24 h of illumination, the relative transcript levels of PhANGs were mildly decreased in *chlm* and *chl27* mutants, whereas those of PhAPGs were not diminished compared to the wild type (**Fig. 3A**). On the other hand, the transcriptional levels of both PhANGs and PhAPGs were overall lower in +DEX *amiR-MGD1* seedlings than in the DEX-untreated control. These results suggest the particular importance of galactolipid biosynthesis in the expression of PhANGs and PhAPGs during chloroplast development.

We previously reported that the DGDG-deficient mutant *dgd1-1* also showed retarded chloroplast differentiation from etioplasts (Fujii et al. 2019). To test if the suppression of MGDG biosynthesis and DGDG biosynthesis has a differential impact on the expression of PhANGs and PhAPGs, we measured the mRNA level of seven photosynthesis-associated genes in *dgd1-1* seedlings illuminated for 24 h after 4 d of etiolation (**Fig. 3B**). Besides *RBCS1A*, transcript abundance of six genes was reduced in *dgd1-1*. The pattern of relative transcript levels was similar between *dgd1-1* and DEX-treated *amiR-MGD1*, indicating that the biosynthesis of MGDG and DGDG has a comparable influence on the expression of PhANGs and PhAPGs.

### Mutation of *GUN1* attenuated the downregulation of PhANGs in MGDG-deficient seedlings

**Fig. 4:**
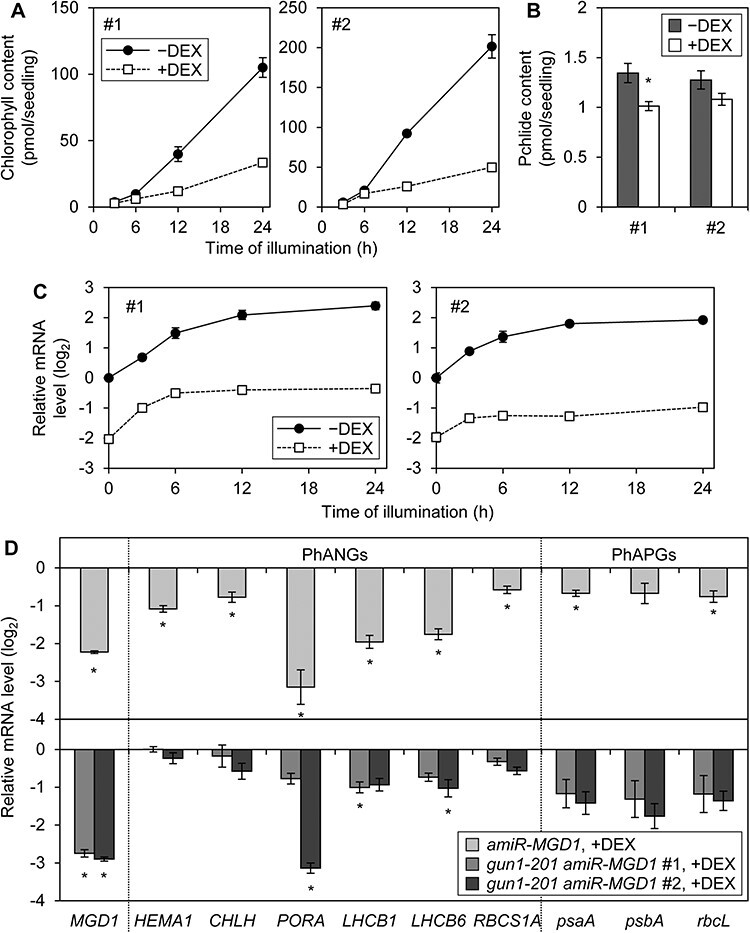
Influence of GUN1 in the de-etiolation process of *MGD1*-suppressed seedlings. (A) Chlorophyll accumulation during de-etiolation in *gun1-201 amiR-MGD1* lines. Seedlings were illuminated under continuous light for the indicated time length after 4-day growth in the dark. Data are means ± SE from eight biological replicates. (B) Protochlorophyllide accumulation in 4-day-old etiolated seedlings of *gun1-201 amiR-MGD1* lines. Data are means ± SE from 12 biological replicates. An asterisk indicates statistical significance (*P* < 0.05, Student’s *t*-test). (C) RT-qPCR analyses of *MGD1* in *gun1-201 amiR-MGD1* lines. Seedlings were illuminated under continuous light for the indicated time length after 4-day growth in the dark. Transcript levels were normalized to *ACTIN8* and presented as the difference from the control before illumination. Data are means ± SE from three biological replicates. (D) RT-qPCR analyses of PhANGs and PhAPGs in *gun1-201 amiR-MGD1* lines. Seedlings were illuminated under continuous light for 24 h after 4-day growth in the dark. Transcript levels were normalized to *ACTIN8* and presented as the difference from each corresponding DEX-untreated control. Data are means ± SE from three biological replicates. Asterisks indicate statistical significance (*P* < 0.05, Student’s *t*-test). Data of *amiR-MGD1* were adapted from a previous article (Fujii et al. 2019) and **Fig. 1** for comparison. In (A)–(D), +DEX indicates seedlings supplemented with DEX from germination until being harvested, whereas −DEX indicates non-treated controls.

GUN1 is a key regulator for the downregulation of PhANGs in response to the impairment of chloroplast functions (Pesaresi and Kim 2019, Shimizu and Masuda 2021, Wu and Bock 2021). To address the involvement of GUN1 in the downregulation of PhANG expression in *amiR-MGD1* seedlings, we introduced the loss-of-function mutation of *GUN1* (*gun1-201*) in *amiR-MGD1* transgenic lines. Two GUN1-less *amiR-MGD1* lines (*gun1-201 amiR-MGD1*), #1 and #2, showed a pale-green phenotype with retarded chlorophyll accumulation under DEX treatment (**Fig. 4A** and **Supplementary Fig. S2**). Etiolated seedlings of line #1 also showed decreased protochlorophyllide accumulation by DEX treatment (**Fig. 4B**), indicating that the chlorophyll biosynthesis pathway is disturbed in *gun1-201 amiR-MGD1* as in the *amiR-MGD1* single line (Fujii et al. 2017, 2019). DEX treatment suppressed the expression of *MGD1* throughout the de-etiolation process in both lines. Inhibition of light-dependent induction of *MGD1* expression in DEX-treated seedlings was slightly stronger in line #2 than in line #1.

In these lines, we tested the transcript level of six PhANGs (*HEMA1, CHLH, PORA, LHCB1, LHCB6* and *RBCS1A*), which were all downregulated in the *amiR-MGD1* single line (**Fig. 1B**) (Fujii et al. 2019). In both *gun1-201 amiR-MGD1* lines, DEX treatment had small or no impacts on the expression of *HEMA1, CHLH* and *RBCS1A* at 24 h of illumination (**Fig. 4D**). The expression of *PORA* and *LHCB6* was almost unaffected by DEX treatment in line #1, but line #2 showed the decreased mRNA accumulation of *PORA* and *LHCB6* in response to the DEX treatment, presumably because the DEX-induced suppression of *MGD1* expression was stronger in line #2.

**Fig. 5:**
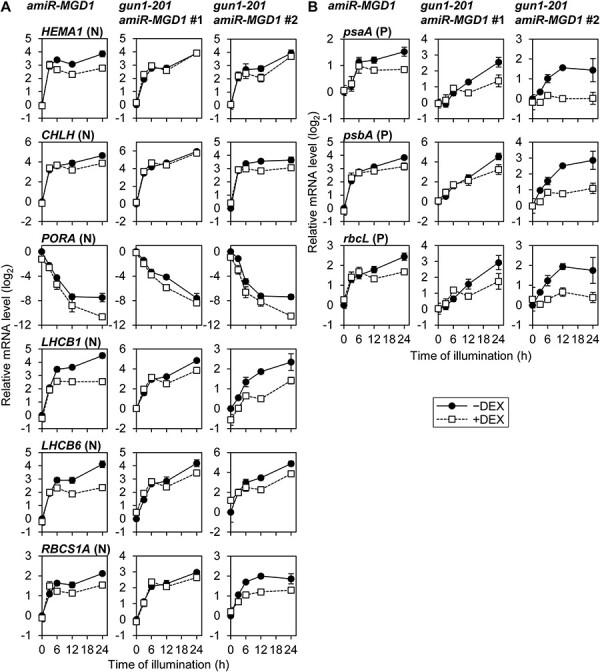
Time course analyses of mRNA accumulation in *gun1-201 amiR-MGD1* lines. (A) RT-qPCR analyses of PhANGs (N). (B) RT-qPCR analyses of PhAPGs (P). In (A) and (B), seedlings were illuminated under continuous light for the indicated time length after 4-day growth in the dark. Transcript levels were normalized to *ACTIN8* and presented as the difference from each corresponding control before illumination. Data are means ± SE from three biological replicates. Data of *amiR-MGD1* were adapted from a previous article (Fujii et al. 2019) and **Fig. 1B** for comparison.

In the DEX-treated *amiR-MGD1* single line, the light-induced mRNA accumulation of *HEMA1, CHLH, LHCB1, LHCB6* and *RBCS1A* was attenuated or halted after 6 h of illumination as reported previously (Fujii et al. 2019) or shown in **Fig. 1B**. To address how GUN1-mediated signaling is involved in the suppression of PhANGs in response to impaired MGDG biosynthesis, we examined temporal changes in transcript levels of these five PhANGs and *PORA* during illumination to etiolated seedlings of the *gun1-201 amiR-MGD1* lines (**Fig. 5A**). In both double lines, the mRNA levels of all PhANGs except *PORA* were acutely increased during the first 6 h of illumination regardless of DEX treatment. Unlike the *amiR-MGD1* single line, the *gun1-201 amiR-MGD1* lines showed continuous increases in the mRNA levels of light-inducible PhANGs after 6 h of illumination regardless of DEX treatment, although the mRNA accumulation of *CHLH* and *RBCS1A* was arrested in line #2. The decreasing pattern of *PORA* transcript levels in the double line #2 was similar to those in *amiR-MGD1* single line, but such suppression was not found in the double line #1. These data suggest the involvement of GUN1 in triggering the attenuation of PhANG expression within 6 h of illumination in seedlings with impaired MGDG biosynthesis.

We also investigated the transcript levels of three PhAPGs (*psaA, psbA, rbcL*) in *gun1-201 amiR-MGD1* lines. Both #1 and #2 lines showed decreased mRNA accumulation of these PhAPGs in response to the DEX treatment at 24 h of illumination (**Fig. 4D**). Time course analysis revealed that, in *gun1-201 amiR-MGD1* lines, the suppression of *MGD1* resulted in attenuation of PhAPG transcript accumulation after 6 h of illumination (**Fig. 5B**). Of note, DEX-treated line #2 showed stronger suppression of PhAPGs than line #1 particularly during the first several hours of illumination, which may be related to the weaker light induction of *MGD1* in line #2 than line #1 in the presence of DEX.

Our results suggest that the *gun1-201* mutation attenuated the *amiR-MGD1*-dependent decreases in the PhANG expression but not PhAPG expression. However, the ecotypes of the *gun1-201* mutant and the *amiR-MGD1* transgenic line were Columbia and Landsberg *erecta*, respectively, so the milder downregulation of PhANGs in *gun1-201 amiR-MGD1* lines than in the *amiR-MGD1* single line might be attributed to the different ecotypes. To test this possibility, we analyzed the transcriptional levels of three PhANGs (*HEMA1, LHCB1* and *RBCS1A*) and three PhAPGs (*psaA, psbA* and *rbcL*), as well as *MGD1*, in the F2 generation of *gun1-201 amiR-MGD1* lines, which were expected to be heterozygous for both the *gun1-201* mutation and the *amiR-MGD1* transgene (**Supplementary Fig. S3**, indicated as *GUN1*^+/−^  *amiR-MGD1*^+/−^), together with the double homozygous lines of the F3 generation. In these double heterozygous lines, the transcriptional pattern of PhANGs and PhAPGs was similar to that in the single homozygous *amiR-MGD1* line, whereas the mRNA levels of PhAPGs in the heterozygous lines were lower than those of the double homozygous plants. These data demonstrate that the ecotype has negligible impacts on the regulation of *MGD1* and photosynthesis-associated genes and indicate the specific involvement of GUN1 in the downregulation of PhANGs in response to *MGD1* suppression.

### Abundance of photosynthesis-associated proteins in the early phase of de-etiolation

**Fig. 6:**
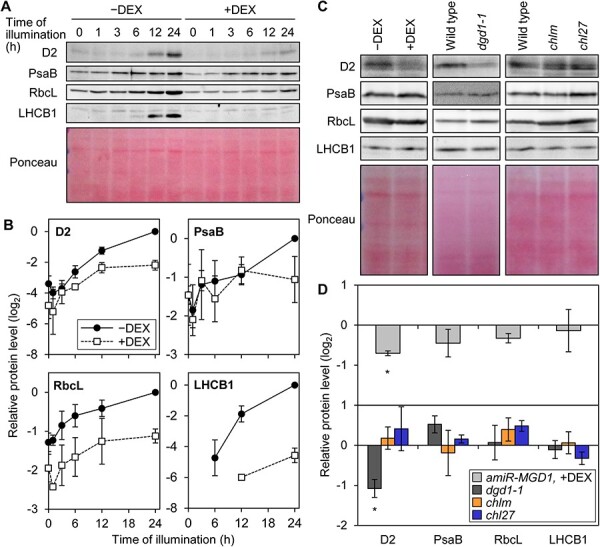
Accumulation of photosynthesis-associated proteins during de-etiolation. (A) Immunoblot analyses in *amiR-MGD1* seedlings. Seedlings were illuminated under continuous light for the indicated time length after 4-day growth in the dark (0 h). (B) Quantification of chemiluminescence signals in the time course immunoblot analysis. Data are means ± SE of signal intensities from three biological replicates after being normalized to the control of each series at 24 h of illumination. In (A) and (B), data of LHCB1 were adapted from a previous article (Fujii et al. 2019). Signals were undetectable in the −DEX samples illuminated for 3 h or less and +DEX samples illuminated for 6 h or less. (C) Immunoblot analyses of photosynthesis-associated proteins in etiolated *amiR-MGD1, dgd1-1, chlm* and *chl27* seedlings illuminated for 3 h. (D) Quantification of chemiluminescence signals in (C). Data are means ± SE of signal intensities from three biological replicates after being normalized to each control (wild type or −DEX control). Asterisks indicate statistical significance (*P* < 0.05, paired *t*-test with Bonferroni correction). In (A)–(D), +DEX indicates *amiR-MGD1* seedlings supplemented with DEX from germination until being harvested, whereas −DEX indicates non-treated controls. In (A) and (C), representative data from three biological replicates are shown. Ponceau S-stained membranes between 25 and 75 kDa are shown as loading controls.

GUN1-dependent downregulation of PhANGs is induced by lincomycin treatment, which inhibits translation in plastids (Susek et al. 1993). We hypothesize that suppression of PhANGs in response to galactolipid deficiency may be attributed to decreased protein synthesis in plastids. To test this hypothesis, we profiled the accumulation levels of photosynthesis-associated proteins encoded in the plastid genome, namely D2, PsaB and RbcL, in *amiR-MGD1* seedlings during the process of de-etiolation (**Fig. 6A, B**). The abundance of these plastid-encoded proteins increased by light illumination in DEX-untreated seedlings. In *MGD1*-suppressed samples, the D2 protein level was also elevated by light illumination, but its accumulation was lower than in the control from the stage of 6 h of illumination. Suppression of *MGD1* expression decreased the accumulation of RbcL throughout the de-etiolation process but had a limited impact on PsaB protein levels.

In *amiR-MGD1*, the DEX-dependent difference in mRNA levels of PhANGs became obvious at 6 h of illumination but was not found at 3 h (**Fig. 1A**) (Fujii et al. 2019). We previously analyzed the LHCB1 protein accumulation during de-etiolation (Fujii et al. 2019). By quantifying the signal intensities of these previously obtained data, we found that the protein levels of LHCB1 were below the detection threshold at 3 h of illumination even in the DEX-untreated controls (**Fig. 6A, B**). Thus, we assessed the amount of four photosynthetic proteins at 3 h of illumination in *amiR-MGD1* seedlings as well as in *dgd1-1, chlm* and *chl27* mutants by loading the larger amount of proteins for immunoblot analysis (**Fig. 6C, D**). The relative abundance of D2 protein was attenuated in *MGD1*-suppressed *amiR-MGD1* seedlings compared to the DEX-untreated control and *dgd1-1* seedlings compared to the wild type, but was comparable between chlorophyll-deficient mutants and the wild type. In this experiment, we did not detect a significant difference in the relative protein abundance of PsaB, RbcL and LHCB1 in all lines tested compared to their corresponding controls.

## Discussion

### Importance of galactolipid biosynthesis in induction of photosynthesis-associated gene expression

In this study, we revealed that decreased biosynthesis of MGDG or DGDG represses accumulation of the photosynthesis-associated mRNA during the de-etiolation process (**Figs. 1B**, **3**). The accumulation of some plastid-encoded proteins, namely, D2 and RbcL, was attenuated from the initial phase of de-etiolation in galactolipid-deficient seedlings (**Fig. 6**). By contrast, knock-down mutation of *CHLM* and *CHL27* had a milder impact on PhANGs during de-etiolation (**Fig. 3A**), consistent with previous studies analyzing seedlings of the same mutants (Mochizuki et al. 2008) and miRNA-mediated inducible knock-down lines (Schlicke et al. 2014). Our results also represent no decreases in the PhAPG expression level in these chlorophyll mutants (**Figs. 3A**, **6C, D**). The stronger suppression of the expression of photosynthesis-associated genes, particularly that of PhAPGs, in galactolipid biosynthesis mutants than chlorophyll biosynthesis mutants with similar color phenotypes implies a specific involvement of galactolipid biosynthesis in the expression of photosynthesis-associated genes, especially those in the plastid genome (**Fig. 3**).

We should note that the *amiR-MGD1* line is Landsberg *erecta* ecotype, whereas all other mutants are Columbia ecotypes. To minimize any problems caused by this difference, we used corresponding controls for each line and mutant in all experiments (e.g. DEX-untreated seedlings for *amiR-MGD1* and *gun1-201 amiR-MGD1* and Columbia wild-type plants for other mutants). The similar transcription pattern in the *amiR-MGD1* line (Landsberg *erecta* ecotype) and double heterozygous *GUN1*^+/−^  *amiR-MGD1*^+/−^ lines (**Supplementary Fig. S3**) indicates that the influence of the ecotypic difference is limited at least under our conditions. Although the differences between *amiR-MGD1* and other mutants must be interpreted very carefully, we conclude that our results would demonstrate the importance of galactolipids in the regulation of PhANGs and PhAPGs.

Total galactolipid abundance in de-etiolated *MGD1*-suppressed *amiR-MGD1* and *dgd1-1* is 30 and 39% of each control, respectively (Fujii et al. 2019). The lipid composition and internal membrane structure were differently affected in these two lines (Fujii et al. 2019). Suppression of *MGD1* expression strongly impaired MGDG biosynthesis and mildly affected DGDG biosynthesis, leading to a decrease in MGDG-to-DGDG ratio, whereas *DGD1* mutation mainly retarded DGDG biosynthesis, resulting in a high MGDG-to-DGDG ratio. *MGD1* suppression inhibited the formation of grana stackings but did not affect prolamellar body (PLB)-to-thylakoid transition during de-etiolation (Fujii et al. 2019). By contrast, both the transformation of PLBs to thylakoids and grana development were attenuated in *dgd1-1* (Fujii et al. 2019). Our transcriptional analyses revealed a similar decrease in the expression of PhANGs and PhAPGs in *amiR-MGD1* and *dgd1-1* despite the large difference in galactolipid composition and membrane structure (**Fig. 3B**), suggesting that the transcriptional regulation of photosynthesis-associated genes is linked to the amount of total galactolipid content, but is independent of the galactolipid composition or internal membrane structure.

Impaired transformation from PLB-to-thylakoid was also observed in the overexpressing line of CURVATURE THYLAKOID1A (CURT1A) (Sandoval-Ibáñez et al. 2021), which is an isoform of CURT1 proteins involved in the regulation of the thylakoid structure (Pribil et al. 2014). However, the aberrant PLB-to-thylakoid transition in the CURT1A-overexpressing line did not affect the expression of both PhANGs and PhAPGs (Sandoval-Ibáñez et al. 2021), supporting our conclusion that the thylakoid structure itself is not a determinant of the expression levels of photosynthesis-associated genes.

### PhAPG expression requires galactolipid biosynthesis during chloroplast differentiation

**Fig. 7:**
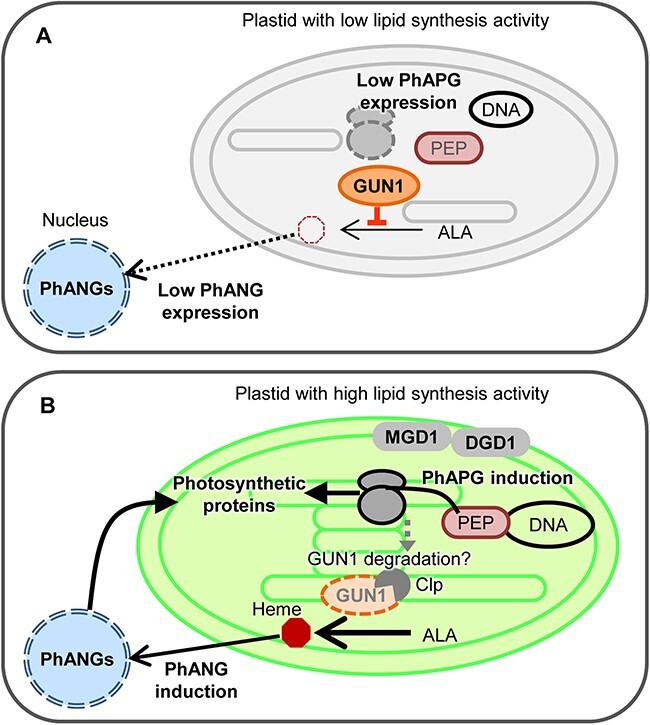
Hypothetical model for coordination of galactolipid biosynthesis and expression of PhAPGs and PhANGs. (A) In plastids with low galactolipid synthesis activity, transcription and translation of PhAPGs are not induced and the accumulation of GUN1 proteins might be prominent. GUN1 may repress heme accumulation and keep PhANG expression at low levels. (B) The increase in galactolipid synthesis activity is followed by the structural change of plastid nucleoids and induction of transcription and translation of PhAPGs. In this condition, Clp proteases might decrease the abundance of GUN1 proteins, possibly resulting in increased production of heme from ALA. Heme is known to upregulate the expression of PhANGs. Expressed photosynthetic proteins contribute to chloroplast development together with accumulated galactolipids. PEP, plastid-encoded RNA polymerase.

Although PhANGs were downregulated in MGDG-deficient seedlings, transcript levels of *rpoB, SIG2* and *SIG6*, which encode proteins required for transcription of PhAPGs including *psaA, psbA* and *rbcL*, were unchanged (**Fig. 1B**). These results suggest that the decrease in the PhAPG expression is not caused by the downregulation of genes required for PEP activity. Similar to galactolipid deficiency, loss of plastid PG biosynthesis specifically suppressed the accumulation of PEP-dependent transcripts without decreasing *rpo* genes (Fujii et al. 2022), suggesting that activation of PEP might be inhibited by impairment of thylakoid lipid biosynthesis regardless of the lipid classes. There might be a tight relationship between membrane lipid synthesis and/or the formation of the thylakoid membrane and PEP functionality in developing chloroplasts (**Fig. 7**). PEP is associated with the plastid membranes at least in young chloroplasts (Finster et al. 2013), so we assume that membrane anchoring of PEP during chloroplast development is important for transcriptional activation of PhAPGs. Since the morphology of plastid nucleoids is also affected in MGDG- and PG-deficient plants (Kobayashi et al. 2013, 2015) and the compaction of plastid nucleoid DNA is associated with transcriptional activity (Sekine et al. 2002), the structure of plastid DNA may also connect the behavior of PEP and membrane lipid biosynthesis.

We detected the suppression of protein accumulation of D2 and RbcL prior to the downregulation of transcript levels of PEP-transcribed genes in DEX-treated *amiR-MGD1* seedlings (**Fig. 6**). Thus, the downregulation of transcription and post-transcriptional processes induced by the impairment of galactolipid biosynthesis may be independent of each other at least at the initial stage of chloroplast development. It remains elusive which processes of protein accumulation in plastids were affected by the loss of galactolipids. One possibility is that galactolipid deficiency destabilizes photosynthesis-associated proteins such as D2 because galactolipid molecules are bound to the PSII complex including D2 protein as structural components (Suga et al. 2015, Su et al. 2017, Shen et al. 2019, Yoshihara et al. 2022). However, considering that the abundance of stromal RbcL proteins is also decreased in MGDG-deficient seedlings, overall protein synthesis is likely to be affected by galactolipid biosynthesis (**Fig. 7**). In *Chlamydomonas reinhardtii*, the dihydrolipoyl acetyltransferase subunit (DLA2) of the chloroplast pyruvate dehydrogenase complex (cpPDC) has an intrinsic RNA-binding activity (Bohne et al. 2013). cpPDC catalyzes the oxidative decarboxylation of pyruvate to acetyl-CoA, the initial reaction in chloroplast fatty acid biosynthesis (Mooney et al. 2002). Although DLA2 is included in fractions with high pyruvate dehydrogenase activity under photoautotrophic conditions, DLA2 is found in a membrane-associated ribonucleoprotein particle under acetate-supplemented mixotrophic conditions, where acetate can be utilized as a substrate of fatty acid biosynthesis (Bohne et al. 2013). DLA2 was shown to be involved in the accumulation of *psbA* mRNA to regions called the translation zone and promotes the D1 synthesis during de novo PSII biogenesis under mixotrophic conditions (Bohne et al. 2013, Neusius et al. 2022), suggesting that DLA2 may coordinate lipid and protein syntheses in *Chlamydomonas* chloroplasts. These data implicate the involvement of several mechanisms in the coordination of membrane lipid synthesis and transcription and translation of PhAPGs during the thylakoid membrane biogenesis. Future studies will elucidate the impact of galactolipid biosynthesis and membrane formation in each phase of transcriptional and post-transcriptional regulation, namely, transcriptional initiation, elongation, RNA stability, RNA processing, translational activity, stabilization of proteins and efficient membrane insertion of proteins, for instance.

### GUN1 is involved in coordination of plastid galactolipid biosynthesis and PhANG expression

Our qPCR analyses revealed that the loss of GUN1 in *MGD1*-suppressed seedlings attenuates the downregulation of PhANGs (**Figs. 4D**, **5**). These results suggest that impairment of galactolipid biosynthesis in plastids may provoke GUN1-mediated plastid signaling to suppress the expression of PhANGs. We previously demonstrated that the downregulation of PhANGs in PG-deficient seedlings is caused by the decreased expression of the transcription factor gene *GLK1* (Fujii et al. 2022). Considering that *GLK1* expression is regulated under the GUN1-mediated plastid signaling (Tokumaru et al. 2017), the loss of plastidic PG may induce the GUN1-dependent signaling pathway and subsequently downregulate the expression of *GLK1*. It is possible that the deficiency of galactolipid and PG biosynthesis downregulates PhANG expression via the same or partially overlapping mechanisms.

Homeostasis of plastid proteins and heme is known to alter the functionality of the GUN1-dependent plastid signaling pathway (Shimizu and Masuda 2021, Wu and Bock 2021). GUN1-dependent downregulation of PhANGs was observed at 6 h of illumination in DEX-treated *amiR-MGD1* seedlings (**Figs. 1B**, **5**) (Fujii et al. 2019), whereas mRNA levels of PhAPGs were not affected by DEX treatment at this timing (**Fig. 1B**). These observations suggest that suppression of PhANGs is independent of the decrease in mRNA abundance in plastids. By contrast, the abundance of some plastid-encoded proteins was decreased already at 3 h of light illumination in *MGD1*-suppressed seedlings and *dgd1-1* seedlings (**Fig. 6**). Inhibition of plastid translation with lincomycin treatment is known to induce GUN1-dependent downregulation of PhANGs (Susek et al. 1993). A recent study suggests that the accumulation of GUN1 in plastids is induced when expression of Clp proteases including plastid-encoded ClpP1 is impaired (Wu et al. 2018). Moreover, *GUN1* mutation causes a strong impairment of chloroplast development in the mutant of plastidic translation initiation factor FUG1 (Marino et al. 2019), indicating that the functionality of GUN1 becomes prominent when plastid protein homeostasis is disturbed. Based on these findings, we assume that the deficiency of galactolipid biosynthesis first impairs the plastid protein homeostasis and thereby induces GUN1-mediated suppression of PhANG expression during de-etiolation (**Fig. 7**). The GUN1 protein level was shown to be relatively high in the beginning phase of chloroplast development or in etiolated seedlings and downregulate the expression of PhANGs probably by regulating the expression of transcription factors including GLK1 (Wu et al. 2018, Hernández-Verdeja et al. 2022). In the course of chloroplast development, the abundance of GUN1 is gradually diminished and the expression of PhANGs is upregulated (Wu et al. 2018, Hernández-Verdeja et al. 2022). In this context, our data indicate the possible involvement of galactolipid biosynthesis in the regulation of GUN1 protein homeostasis (**Fig. 7**). Revealing the relationship between lipid biosynthesis and GUN1 accumulation would be an important goal for future studies.

In addition to plastid protein homeostasis, the level of heme production is known to affect PhANG expression (Woodson et al. 2011, Page et al. 2020). Because ferrochelatase for conversion of Proto IX to heme is localized in the plastid membrane (Espinas et al. 2016), heme biosynthesis may be impaired in the lipid biosynthesis mutants. In this hypothetical scenario, the mutation of *GUN1* in these mutants may elevate the heme accumulation and subsequently compensate for the downregulation of PhANGs, because the loss of GUN1 is known to increase the heme content (Shimizu et al. 2019). The heme homeostasis might depend on galactolipid biosynthesis and optimize the expression level of PhANGs to ensure the coordinated formation of the thylakoid membrane (**Fig. 7**).

The accumulation of nuclear-encoded LHCB1 proteins was downregulated at the stage of 6 h of illumination by *MGD1* suppression, but not altered at 3 h when the transcript level of the *LHCB1* gene was not affected (**Fig. 6C, D**) (Fujii et al. 2019). This result indicates that the lower abundance of LHCB1 protein in galactolipid-deficient seedlings may be initially caused by the downregulation of its transcript level, but is not due to the regulation of translation nor the protein stability on the membrane. This finding also underlines the importance of GUN1-dependent signaling in coupling the galactolipid biosynthesis and PhANG expression during chloroplast biogenesis (**Fig. 7**).

## Conclusion

Our results highlight the contribution of galactolipid biosynthesis in the expression of both PhANGs and PhAPGs. Galactolipids are required for both transcription and post-transcriptional regulation in plastids. GUN1 plays a pivotal role in coordinating galactolipid biosynthesis in plastids and gene expression in nuclei. Based on our findings and previous studies, homeostasis of plastid proteins and/or heme is likely to be important for the GUN1-mediated orchestration of plastid lipid synthesis and PhANG expression.

## Materials and Methods

### Plant materials and growth conditions

*Arabidopsis thaliana* mutants used in this study, namely *chlm, chl27* (Bang et al. 2008, Mochizuki et al. 2008), *dgd1-1* (Dörmann et al. 1995) and *gun1-201* (Martín et al. 2016), were of Columbia ecotype, whereas the *amiR-MGD1* transgenic line (L4w) (Fujii et al. 2014, 2017) was of Landsberg *erecta* ecotype. Surface-sterilized seeds were incubated at 4°C for 3 or 4 d in the dark and grown on an agar-solidified 1× Murashige and Skoog medium containing 1% (w/v) sucrose. To synchronize germination, sown seeds were illuminated under the room light for ∼3 h and then incubated at 23°C for 4 d in complete darkness. Seedlings were then illuminated under continuous white light (50 µmol photons m^−2^ s^−1^) at 23°C for the indicated time length. To induce the expression of *amiR-MGD1* transgene, DEX (FUJIFILM Wako Pure Chemical, Osaka, Japan) was added to a final concentration of 10 µM in the medium from a 50 mM stock in dimethyl sulfoxide. We previously confirmed that the phenotypes between Landsberg *erecta* seedlings and DEX-untreated *amiR-MGD1* seedlings were comparable (Fujii et al. 2014). In each experiment, corresponding control samples, namely, Columbia wild type for *chlm, chl27* and *dgd1-1* mutants and DEX-untreated controls for DEX-treated *amiR-MGD1* and *gun1-201 amiR-MGD1* lines, were grown in parallel.

### Construction of *gun1-201 amiR-MGD1* lines

The *gun1-201* mutant was crossed with four different plants of the *amiR-MGD1* transgenic line L4w (Fujii et al. 2017). Seeds of F2 generation from 60 different F1 plants (15 each from four F0 plants) were collected separately and germinated on a BASTA (BASF, Ludwigshafen, Germany)-containing medium to select plants carrying the *amiR-MGD1* transgene. To select *gun1-201* homozygous plants, eight BASTA-resistant plants from each strain were subjected to genotyping by using MightyAmp DNA polymerase (TaKaRa, Kusatsu, Japan) and primers for detection of T-DNA insertion in the *GUN1* locus (**Supplementary Table S1** and **Fig. S2A**). From 84 strains of the F3 generation carrying the homozygous *gun1-201* mutation, two lines (#1 and #2) were selected by the following criteria: (i) all plants showed a green cotyledon phenotype under DEX-untreated conditions; (ii) all plants showed a pale-green cotyledon phenotype homogeneously under DEX-treated conditions; and (iii) all plants are resistant to BASTA (**Supplementary Fig. S2**). Two selected lines of the F3 generation were utilized for the analysis. For the F2 generation, two lines (#1 and #2) were selected by the following criteria: (i) all plants showed a green cotyledon phenotype under DEX-untreated conditions and (ii) at least some seedlings showed a pale-green cotyledon phenotype under DEX-treated conditions. These pale-green seedlings were utilized as DEX-treated seedlings with strong *MGD1* suppression.

### RT-qPCR analysis

Extraction of total RNA, genomic DNA digestion and reverse transcription were conducted as described (Fujii et al. 2014). Quantification of transcript levels was performed as described (Fujii et al. 2014) by using the real-time PCR system Mx3000P (Agilent Technologies, Santa Clara, CA, USA; *dgd1-1* in **Fig. 3B** and **Supplementary Fig. S3**) or CFX96 (Bio-Rad, Hercules, CA, USA; all the other experiments). The relative transcript abundance is shown as the mean of the logarithm of all replicates after being normalized to the level of a reference gene *ACTIN8* (Pfaffl 2001). Gene-specific primers used in this study are listed in **Supplementary Table S2**. For analyses of *psaA, psbA, rbcL, RBCS1A, PORA, rpoB, SIG2, SIG6, RPOTp* and *RPOTmp* in *amiR-MGD1* single lines, we used cDNA samples which had been generated previously and used for the analyses of *MGD1, HEMA1, CHLH, LHCB1* and *LHCB6* (Fujii et al. 2019).

### Chlorophyll determination

Chlorophylls were extracted by incubating whole seedlings in 80% (v/v) acetone at 4°C in darkness for 2 or 3 d. Chlorophyll content was determined by measuring the absorbance at 663 and 645 nm with a V-370 BIO (JASCO, Hachioji, Japan) spectrophotometer as described (Melis et al. 1987) after normalization at 720 nm or measuring fluorescence emission at 666 nm under excitation at 440 nm with an RT-5300PC spectrofluorometer (Shimadzu, Kyoto, Japan) by referring the chlorophyll standard sample of known concentration as described (Fujii et al. 2014).

### Measurement of tetrapyrrole intermediates

To address the state of the chlorophyll biosynthesis pathway in illuminated seedlings, 4-day-old etiolated seedlings were illuminated for 3 h as described earlier. Then, seedlings were incubated in a solution containing 10 mM ALA, 10 mM MES-KOH (pH 5.7) and 5 mM MgCl_2_, with or without 10 µM DEX for 1 h in the dark. Porphyrin intermediates were extracted in *N,N*-dimethylformamide and quantified by HPLC as described (Fujii et al. 2017).

### Immunoblot analysis

Total proteins were extracted from seedlings and quantified by using the RC DC Protein Assay (Bio-Rad) as described (Fujii et al. 2017). A total of 10 (**Fig. 6A, B**) or 50 (**Fig. 6C, D**) µg of total proteins were separated with SDS-PAGE unless otherwise stated and detected as described (Fujii et al. 2017). To detect D2, PsaB, RbcL and LHCB1 in *dgd1-1* and its control, we used 60, 20, 20 and 20 µg of total proteins, respectively. Polyclonal antibodies against D2 (provided by M. Ikeuchi, The University of Tokyo), PsaB, RbcL and LHCB1 (Agrisera, Vännäs, Sweden) were utilized as primary antibodies. The signal intensities were quantified by using ImageJ software.

## Supplementary Material

pcae049_Supp

## Data Availability

The data underlying this article are available in the article and in its online supplementary material. Further information will be shared on reasonable request to the corresponding author.
