# Supplementary material for: Orchestration of Photosynthesis-Associated Gene Expression and Galactolipid Biosynthesis during Chloroplast Differentiation in Plants
Source: Plant Cell Physiol. 2024 Apr 26;65(6):1014–28. doi: 10.1093/pcp/pcae049 (PMC11209550; doi:10.1093/pcp/pcae049)
Supplement: pcae049_Supp [file pcae049_supp.zip › pcae049_Supp/Fujii_et_al_Supplemental_materials_rev2_corrected.pdf]

Supplementary materials

Supplementary Table S1. Oligonucleotide primers used for genotyping.

Name	Sequence (5’ → 3’)
RP	ATGTTGTAACTTATAATTCCATAATCGATGCC
LP	CTATCACAAGAAAACATAGCATCATCAAGGAC
LB	GCTTCCTATTATATCTTCCCAAATTACCAATACA

Supplementary Table S2. Oligonucleotide primers used for reverse transcription-quantitative PCR analysis.

Name	Gene ID	Forward primer (5' → 3')	Reverse primer (5' → 3')
ACT8	AT1G49240	ACTGTGCCTATCTACGAGGGTTTC	CCCGTTCTGCTGTTGTGGT
MGD1	AT4G31780	GCAGGACTTGAAACATCACAAATC	GCGAACTGGTTTCACAAAGGA
HEMA1	AT1G58290	TAAGATTAGCTTCCCCACAAACTC	AGCTCGCTTATAGCTTCACAACAC
CHLH	AT5G13630	TGGTAGAGAGACAGAAGCTCGAAA	CCAAAGAACCTGCCCAAGAG
PORA	AT5G54190	GAGTTTGGGAAGTCAGCGAGA	CAAAGGTTGAAACACCGAGGA
LHCB1.2	AT1G29910	CCCGAGACATTCGCAAGGAACCGTG	AAGCTCAGGGAAGACGCAGCCTAGG
LHCB6	AT1G15820	GGACTTTGAGAAGCTGGAGAGG	ACAAACCAAGAGCACCGAGAG
RBCS1A	AT1G67090	AGATGACCAAAGCACTAGACCAAAC	AGTAGCGGAAGAGAGCATAGAGGA
SIG2	AT1G08540	TTGGACAAAGTGTTGGACTCGT	CTTCATTCTCCCATCCTCCATC
SIG6	AT2G36990	GACGGGCTCCAAAATCAAAC	TCTCAGGTCCCCACAAGAAGA
RPOTp	AT2G24120	AGGGTAACACGGTCGATGTTAGG	GCCTCTCGACATGCAACAGC
RPOTmp	AT5G15700	CAGCATTATGCCGCTCTTGGG	GTCTGCATCTCGGCGCATAATATC
psaA	ATCG00350	GTTAGTAGCAGTGGGTGGCAAAG	TAAACGCGAGCTACGAGCAA
psbA	ATCG00020	GAGAGACGCGAAAGCGAAA	TCATCAAAACACCAAACCATCC
rbcL	ATCG00490	ACTTGAAGGAGACAGGGAGTCAA	TGAAGCCACAGGCAGAACA
rpoB	ATCG00190	CTGGAGGGGTTTGGTGTTG	CGGAGCATCTTCTGGTTTAGGT

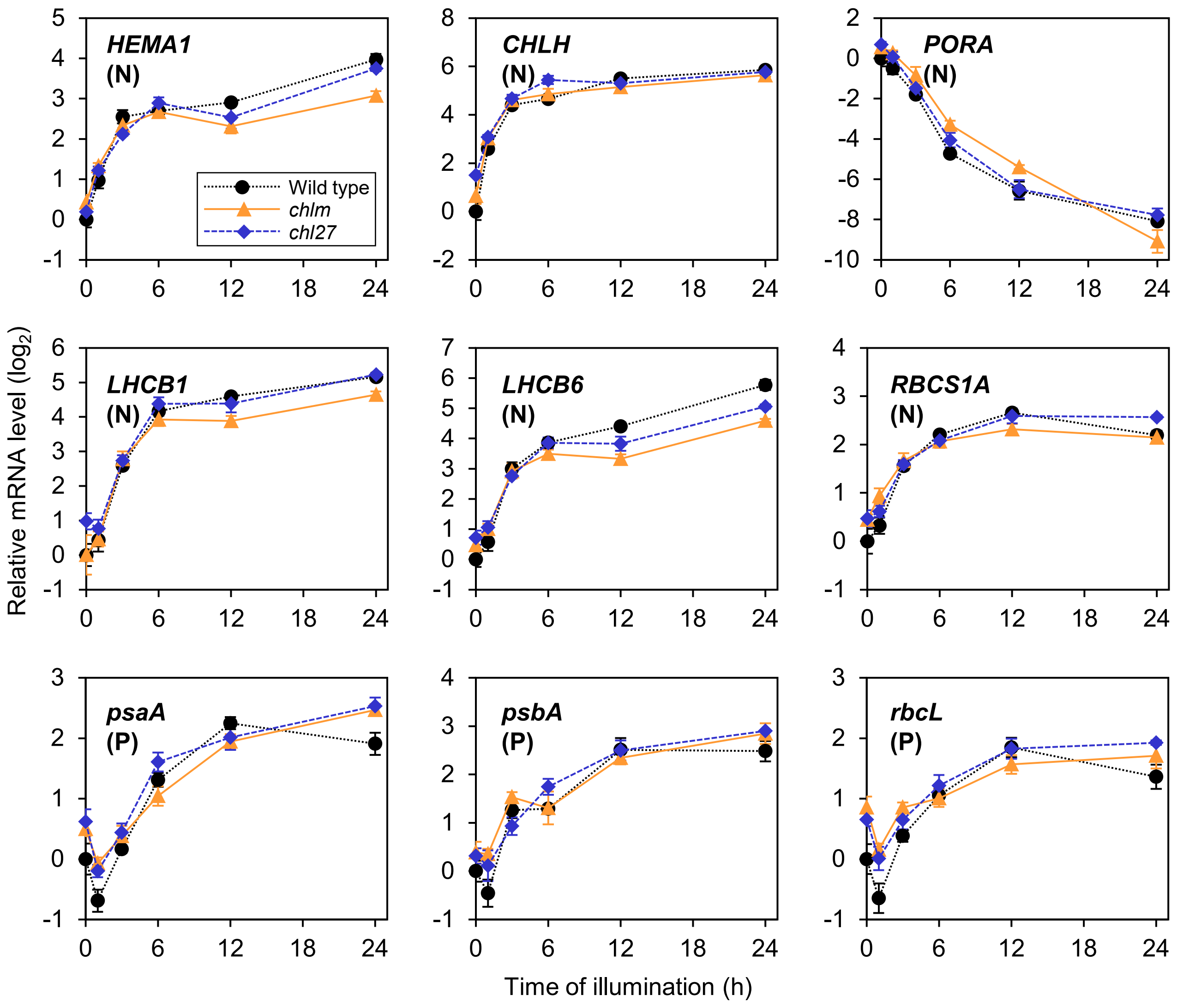

Figure S1. mRNA accumulation of PhANGs and PhAPGs during deetiolation in chlorophyll-deficient seedlings. Seedlings were illuminated under continuous light for the indicated time length after 4-d growth in the dark (0 h). Transcript levels were normalized to *ACTIN8* and presented as the difference from the control before illumination. Data are means ± SE from three biological replicates.

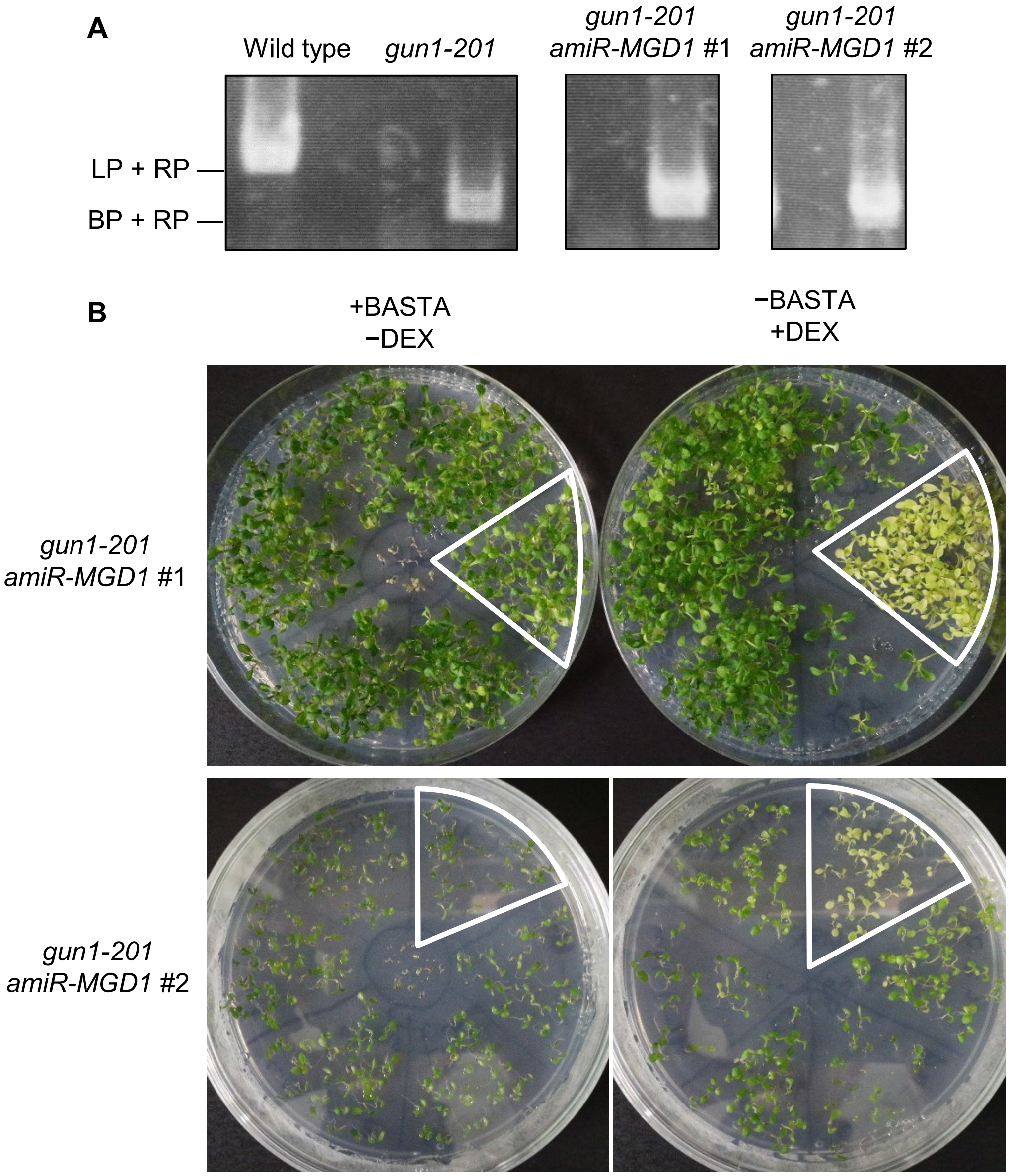

Figure S2. Screening of *gun1-201 amiR-MGD1* lines. A, Genotyping of F2 generation. LP + RP and BP +RP indicate the amplification of the wild-type or T-DNA-inserted *GUN1* gene, respectively. Results of wild type and *gun1-201* homozygous mutants were shown as a control. B, Phenotype of F3 generation. Plants were grown for 10 d (upper) or 5 d (lower) on the medium containing either BASTA or DEX. Lines #1 and #2 are indicated by white frames.

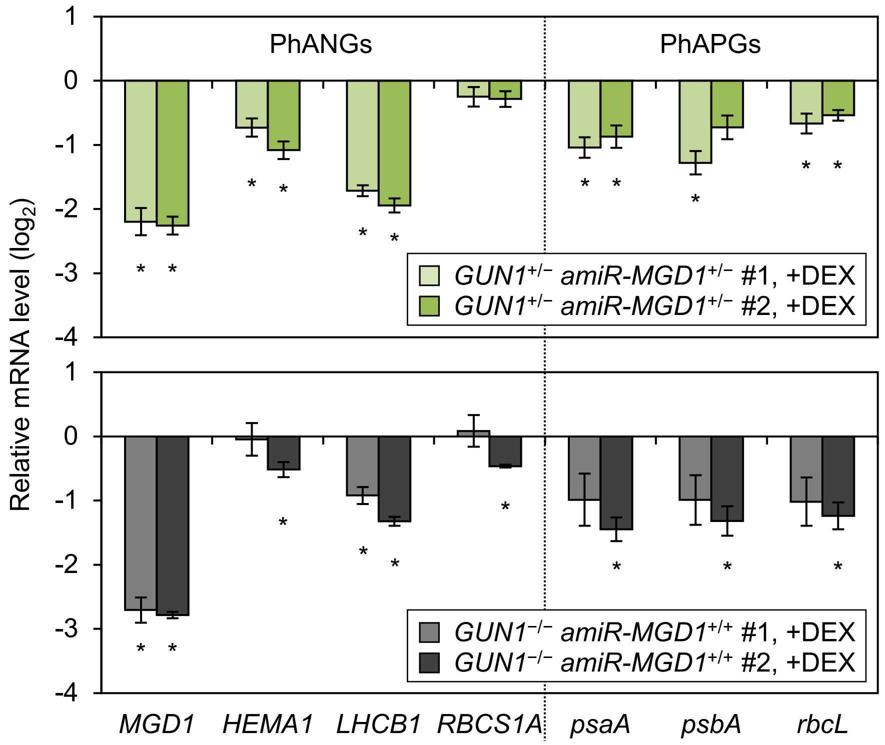

Figure S3. mRNA accumulation of PhANGs and PhAPGs in heterozygous *GUN1*$^{+/-}$ *amiR-MGD1*$^{+/-}$ lines. Seedlings were illuminated under continuous light for 24 h after 4-d growth in the dark. Transcript levels were normalized to *ACTIN8* and presented as the difference from each control. Data are means ± SE from three biological replicates. *GUN1*$^{+/-}$ *amiR-MGD1*$^{+/-}$ lines are selected from the F2 generation whereas *GUN1*$^{-/-}$ *amiR-MGD1*$^{+/+}$ lines (equivalent to *gun1-201 amiR-MGD1*) are from the F3 generation. Asterisks indicate statistical significance ($p < 0.05$, Student's *t*-test).
